# Primary care occupational therapist’s methods of outcome evaluation: Do they align to value-based healthcare?

**DOI:** 10.1177/03080226251320185

**Published:** 2025-02-24

**Authors:** Laura Ingham, Alison Cooper, Catherine Purcell

**Affiliations:** 1 School of Healthcare Sciences, Cardiff University, Cardiff, UK; 2 Swansea Bay University Health Board, Singleton Hospital, Sketty Lane, Swansea, UK; 3 Population Medicine, School of Medicine, Cardiff University, Cardiff, UK

**Keywords:** Occupational therapy, primary care, general practice, evaluation methods, value-based healthcare

## Abstract

**Introduction:**

Occupational therapy roles are increasing across General Practice in primary care. The evidence base is growing; however, the best way to evaluate outcomes and the impact of practice in this setting remains unclear. Consideration for how methods used align to ambitions of value-based healthcare is also required. This study explored evaluation methods used by occupational therapists, providing services to General Practice in Wales within the context of value-based healthcare.

**Method:**

An online focus group was conducted with 13 members of a Welsh Primary Care Occupational Therapy network. Mixed methods were used and Mentimeter results and findings from group discussion were analysed through content and framework analysis.

**Results:**

A multifaceted but inconsistent approach to evaluation was reported. Methods used, strengthened by professional core values, broadly aligned practice to shared ambitions of value-based healthcare. The use of validated patient rated scales were most commonly used to evaluate patient experience, whilst cost-effectiveness was least well considered.

**Conclusion:**

Further research is required to understand occupational therapy evaluation in this setting to identify what is needed by stakeholders to determine impact and establish value. This could inform care at both an individual level and across populations if consistent data are collected at scale.

## Introduction

By 2041, almost 21 million or one in four people within the United Kingdom (UK) will be aged 65 and over (Office for Health Improvement & Disparities, 2023). The associated health and care needs of this trend are rising (Thinley, 2021). To address this, healthcare reform worldwide has adopted a population health focus. Population health aims to keep people well and achieve improved health outcomes through placing greater emphasis on the determinants of health, prevention and health promotion (Atala et al., 2023). Primary care is described fundamentally as the first point of contact to accessing healthcare, supporting continuity, comprehensive and coordinated care (Starfield, 1992). Primary care encompasses population health approaches with ambitions to achieve greater equity within system-wide community-based services (World Health Organization and United Nations Children’s Fund, 2018). A wider skill mix is being seen as instrumental to embedding this approach and supporting health and care provision in primary care (Scottish Government, 2020; Welsh Government, 2020; World Health Organization, 2018) and occupational therapists, who support a person’s participation in meaningful occupations to achieve health whilst addressing wider determinants at both individual and population level, are well situated to provide preventative approaches (Braveman, 2015; Dahl-Popolizio et al., 2018). As a result, occupational therapy roles are being identified in plans for primary care development (Department of Health, 2016; NHS England, 2019; NHS Scottland, 2018). There is evidence that occupational therapy services are becoming embedded in primary care settings (Bolt et al., 2019; Donnelly et al., 2023). This has included within the model of care provision known traditionally as General Practice (GP), or globally termed Family Practice (World Health Organization and United Nations Children’s Fund, 2020), where occupational therapists are considered a sustainable solution to rising workforce and healthcare pressures (Brooks et al., 2017).

In Wales, the Welsh Governments plan: A Healthier Wales has been pivotal to healthcare reform. The Welsh Governments plans: A Healthier Wales has reshaped GP provision towards a cluster-based model of working whereby care is delivered through grouping together traditionally organised GP surgeries and other community services across specific geographical areas (Welsh Government, 2018). Depending on local population need, cluster working has included the integration of an Allied Health Profession (AHP) workforce including occupational therapists, to support initial access to multidisciplinary care for people in the community, alongside other professionals such as GPs, pharmacists and nurses (Primary care One, 2021). To drive quality, reduce healthcare resource waste, reduce variation in service provision and increase higher value care, ‘Prudent principles’ have underpinned this reform. Plans for change in Wales are also centred around the internationally adopted quadruple aims (Welsh Government, 2018). These aims build on the triple aim ambitions of Berwick et al. (2008) to improve population health, increase quality of care through a focus on peoples experiences of the system and increase value, with the introduction of a fourth aim to sustain a suitable workforce, all in the global pursuit of more sustainable healthcare (Bodenheimer and Sinsky, 2014). To achieve a system fit for meeting future population needs, the Welsh Government’s plans have also been strengthened by principles of the internationally expanding approach, value-based healthcare (VBHC; Lewis, 2022). Encompassing quadruple aim ambitions, VBHC aims to optimise healthcare provision using a focus on outcomes that matter most to individuals (Teisberg et al., 2020). Moreover, rather than using primarily performance related outcomes such as clinical outputs or cost-savings, as was traditionally the case, to achieve high value care, the approach prioritises meaningful outcomes relative to costs spent (Porter, 2010). In private-funded systems, analysis of outcomes and costs can drive competition at a pathway level. In more publicly funded models however, such as that in the UK, a more population-based approach places broader emphasis on quality and resource allocation to optimise value (Lewis, 2019). This perspective enables outcome comparison across groups to optimally manage resources to address population health (Bedlington et al., 2021; Gray, 2015) and thus observes VBHC as ‘the equitable, sustainable and transparent use of the available resources to achieve better outcomes and experiences for every person’ (Hurst et al., 2019: 3).

The shared goal of promoting health, by considering people’s outcomes and experiences remain central to all perspectives of the VBHC approach (Hurst et al., 2019; Porter, 2010). Person-centred principles underpin implementation through mechanisms such as shared decision making and identifying goals on which care should be based (Wong et al., 2022) in addition to a range of indicators and evaluation measures used to plan care (Bedlington et al., 2021). Within the value-based approach, these mechanisms include Patient Rated Outcome Measures (PROMs) that enable individuals to establish what matters most through self-reporting on their own health, by using a range of domains and Patient-Rated Experience Measures (PREMs; Withers et al., 2021). These tools inform care provision at an individual level and, supported by data collection at scale, can inform cost-effectiveness and resource allocation across pathways of care and at a population level (Bedlington et al., 2021; Lewis, 2022: Withers et al., 2021).

The principles of VBHC align suitably to ambitions of a reforming model of primary care (Porter et al., 2013) and international uptake of the original triple aim perspective to evaluate improvements in primary care has been shown (Obucina et al., 2018). Occupational therapy, which shares a person-centred focus, is also well placed to contribute to aspirations of achieving higher value healthcare (Lamb and Metzler, 2014; Mroz et al., 2015) and in Wales this ambition is shared and operationalised through a framework to optimise the AHP skillset in the pursuit for achieving better population health (Welsh Government, 2020). At present however, there is a sparsity of evidence aligning the profession to this approach. Wong et al. (2022) gathered stakeholders’ perspectives to explore this. Whilst participants attributed the meaning of value to goals, outcomes, management of personal costs and experiences of healthcare provider relationships, recommendations included an increasing need to consider goals, meaningful outcome measurement and supporting data collection at scale (Wong et al., 2022). Whilst more needs to be done, the findings of this study highlight similarities between VBHC and occupational therapy approaches. This alignment may provide an opportunity to promote how the profession can support health (Braveman, 2015; Lamb and Metzler, 2014); however, without a thorough understanding of how to evidence its value-based contribution, a risk to the development of the profession is posed (Leland et al., 2015; Wong et al., 2022). The same could be said about occupational therapy in primary care given the ongoing need to identify measures and indicators to support development has been highlighted (Donnelly et al., 2017). Currently, only a few studies explore outcome evaluation in this setting (Donnelly et al., 2017; Hand et al., 2022) and subsequently a poor understanding of how best to evaluate and demonstrate the value and impact of the occupational therapy offer in this setting remains (Hand et al., 2022; Letts et al., 2024). Substantiated by a recent survey of practitioners (Royal College of Occupational Therapists, 2023) and as prioritised as an area for research (Royal College of Occupational Therapists, 2021b), the profession appears committed to developing mechanisms to achieve this. Previous research has considered resource use and cost-effectiveness (Cook and Howe, 2003; Lambert et al., 2010; Usher and Connolly, 2018), important denominators for value, however at present evidence for this remains limited. Without more knowledge about the methods of outcome evaluation being used by occupational therapists who support people in primary care as part of GP or Cluster models of working, establishing the value and contribution within the current transformation agenda may be difficult to determine. This could limit opportunities for securing funding to support future development (Letts et al., 2024) and result in inequality of care and misalignment to the prudent ways of working required to address population health (Welsh Government, 2018). To understand what value occupational therapy is contributing in this setting, inform further service development and more firmly embed the profession, a thorough understanding of how evaluation can demonstrate effectiveness at an individual level and capture data at scale to support population health is required. The aim of this study therefore was to conduct a Wales wide review of evaluation methods being used by primary care occupational therapists to evaluate interventions and services being provided within cluster models of working to understand current use and to explore how methods currently align with VBHC.

## Methods

### Research design

As part of a sequential mixed-method study, the initial phase of research is described and is informed by pragmatism, based on an understanding of what works in practice (Patton, 2015). Described by Kelly and Cordeiro (2020) as useful in organisations to support ‘exploring and understanding the connections between knowledge and action in context’ (p. 1) and given the different contexts that may influence evaluation of occupational therapy in primary care, this theoretical stance was deemed suitable.

### Participants

The UK setting of a Wales wide Primary Care Occupational Therapy (PCOT) network was used alongside a criterion-based sampling technique to invite members (occupational therapists and occupational therapy students or unregistered occupational therapy support staff), who work or had recent experience (within the last 2 years) of providing services in primary care clusters in Wales. The appointment of a gatekeeper who distributed information about the study to PCOT network members supported recruitment.

### Data collection

During an online PCOT network group meeting, a 1-hour focus group explored perspectives and experiences of occupational therapists (see Supplemental Information for details of questions). Mentimeter (Mentimeter AB publ), an interactive web-based polling tool, was used for each question and produced word cloud representations, a strategy suggested by deNoyelles and Reyes-Foster (2015) as useful for aiding engagement, alongside the collection of anonymous quantitative and qualitative data. Further discussion was then invited for 8 of the 15 questions to encourage deeper exploration of the Mentimeter responses. A transcript of the discussion was captured using ‘Otter.ai’ software, https://otter.ai/ and once fully pseudo-anonymised, the transcript was emailed to the attendees for member checking which included checking content for accuracy. All data were managed in accordance with research and data protection regulations.

### Data analysis

Quantitative Mentimeter data were analysed descriptively to identify patterns of evaluation in this setting. Content analysis (Domas White and Marsh, 2006) was used to analyse reported intervention types which were coded and through reflection on latent meaning and similarities of the interventions identified, organised into categories to provide insight into current practice. To support a more thorough understanding of occupational therapists’ perspectives and flexibly enable analytical triangulation of both the Mentimeter responses and open discussion data, framework analysis, a rigorous and systematic branch of thematic analysis, was used (Ritchie and Spencer, 1994). Framework analysis is used throughout qualitative healthcare research in cross-sectional data analysis (Goldsmith, 2021) and was therefore deemed appropriate. The data were considered within five stages, with the main aim of identifying an analytic framework consisting of themes and sub-themes for the purpose of applying it to the whole dataset (Goldsmith, 2021; Ritchie and Spencer, 1994; Spencer et al., 2014). Initially Mentimeter responses and field notes taken during the focus group were reviewed during the first stage of data familiarisation. A deductive approach ensued during the second stage of analysis, where priori questions posed in the focus group were used to identify initial categories (or sub-themes) and recurring themes within a framework. Within this, two overarching themes: evaluation methods and influencing factors, were developed. Stage 3 of analysis required indexing and charting, where data from the Mentimeter responses was compared, combined and further refined within the framework (Ward et al., 2013). In stage 4, given the brief responses captured within mentimeter, summarising of data responses was not required; however, when then applied to the whole dataset, which involved charting the open discussion qualitative responses into the draft framework, this stage required condensation of quotes taken verbatim through the focus group discussion. Throughout this later stage and when the discussion data was combined with the Mentimeter responses, a more iterative, inductive approach was employed. During the final mapping and interpretation stage of the whole dataset, refining of themes and sub-themes represented the final conceptual framework. For example, during earlier stages, a theme of ‘OT feelings’ was included; however, during the mapping and interpretation stage, this was removed as the theme appeared closely synonymous within other sub-themes described. The findings presented within the final conceptual framework enabled consideration within a VBHC context to inform the outcomes of the study.

### Rigour

The first author (LI) led the study and implemented a range of mechanisms to support analysis. This included in earlier stages the use of word documents to index and chart data. For later stages, when coding the focus group open discussion data, the computer-assisted software Nvivo (Lumivero, https://lumivero.com/) was used. A range of methods is considered typical in the framework analysis approach (Goldsmith, 2021) and to ensure that the analysis remained rigorous and credible during all stages, reflexive field notes were kept (Ritchie and Spencer, 1994). This practice facilitated regular discussion and email communication with the other authors (AC and CP) to support decisions at all stages.

### Ethics

Approval for this study was obtained on 19th June 2023, and the study was conducted in alignment with the approved protocol. Participants were invited on a voluntary basis and assured of confidentiality as part of the informed written consent process. Due to the small number of primary care occupational therapists in Wales and risk of participant identification, members who took part have not been presented demographically and the seven health boards in Wales have been anonymised.

## Findings

### Participants

**Figure 1.**
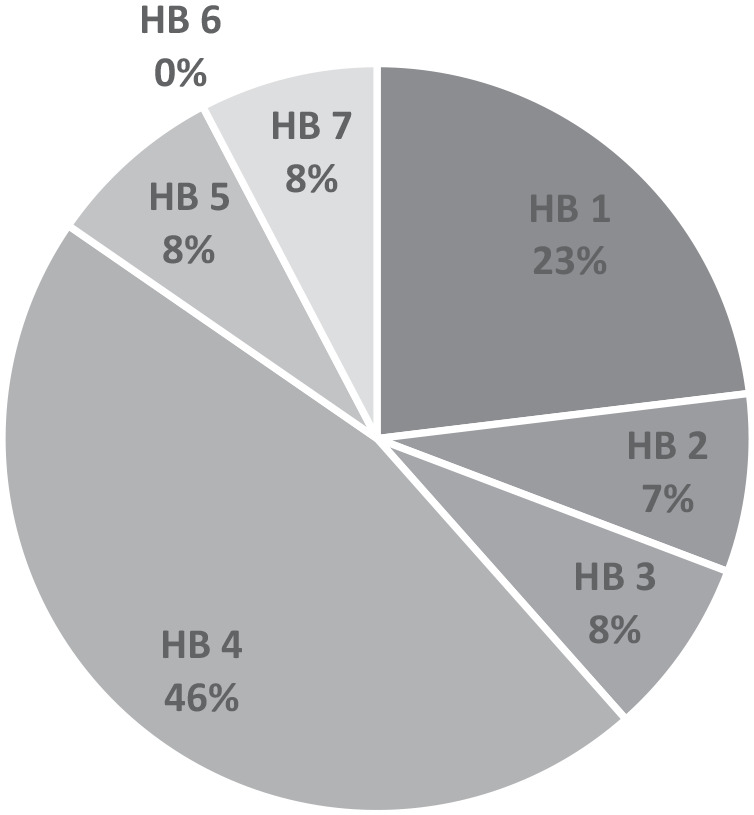
Anonymised health board organisations in Wales represented by participant shown as percentage.

Within the PCOT network group meeting, 13 members attended and consented to participate in the focus group. The participants were all registered practitioners and geographically represented health board organisations across Wales with the exception of one Health Board, who at the time of the discussion was not known to have occupational therapy representation in primary care clusters (see Figure 1). A total of 12 out of 13 participants identified their banding (UK health service pay grade category with higher bands denoting increased skill, level of experience or management responsibility). Two were band six, seven were band seven and three were band eight or above (median band seven). A mean length of service in a PCOT role of 2.4 years was reported (see Table 1).

**Table 1.** Length of service in PCOT role.

No of years	Participants
Under 1	4
1–2	1
2–4	5
4–6	0
Over 6	2
	Mean = 2.4

PCOT: primary care occupational therapy.

### Service provision

**Figure 2.**
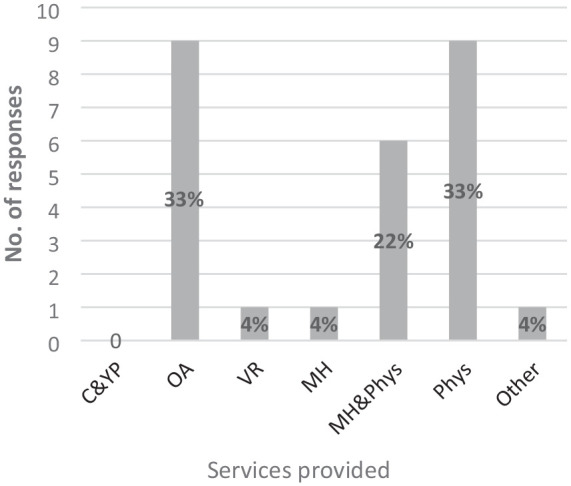
Type of service provided in setting. C&YP: children & young people; OA: older adults; VR: vocational rehab; MH: mental health; MH&PH: mental health & physical health; Phys: Physical Health; Other.

Participants were asked to identify the type of services provided in their setting (see Figure 2). Of the 12 out of 13 participants who responded, all were working with adults only and nine participants (33% of responses) identified they were providing physical health services. Similarly, nine (33%) were providing services to older adults and a combination of physical and mental health services were reported by six respondents (22%). Of note is that 10 of the 12 participants (83%) who responded to this question reported provision of a range of services (see Table 2), one participant reported a combination of services in physical or mental health, four participants (33%) reported services to older adults with a physical health focus and five participants (42%) identified a combination of physical and mental health services, physical health and older adults. The provision of vocational rehabilitation was reported once (4%) and only two participants noted either mental health or physical health service provision exclusively.

**Table 2.** Combination of service provision reported by participants.

Participant	Children and young people	Older adults	Vocational rehab	Mental Health	Mental Health and Physical	Physical	Other
1		x			x	x	
2						x	
3				x			
4		x				x	
5		x				x	
6		x			x	x	
7		x			x	x	
8		x	x		x		
9					x		
10		x			x	x	
11		x				x	
12		x				x	

**Figure 3.**
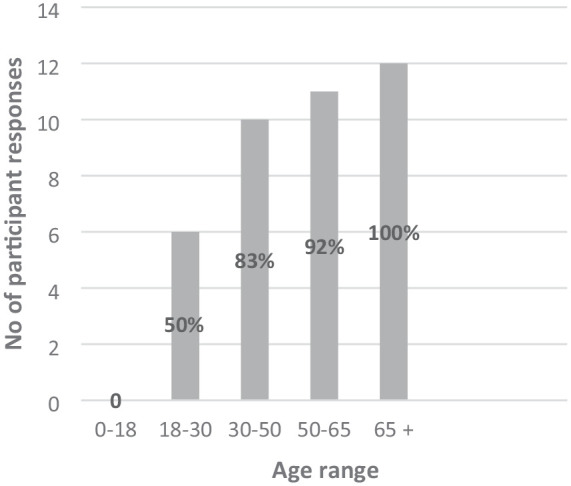
Age ranges that participants typically work with.

Participants identified working across the lifespan with the exception of working with children and young people. Out of the participants who provided responses, 50% reported working with those aged 18–30 years, 10 responses indicated 83% of the participants worked with those aged 30–50 years, 11 responses indicated that 92% of the participants worked with adults aged 50–65 years and all participants (100%) reported providing services to those aged 65 years and over (see Figure 3).

Participants were asked to identify up to five interventions typically used in their setting. The responses collected were coded and categorised and frequency of both of these noted, further details available in Supplemental Information. Self-management, symptom management was the most frequently cited category identified and reported as an intervention used across services for adults of all ages by one participant working specifically in mental health and by all participants who described providing services in both physical and mental health, four of which with older adults. The category of an intervention focusing on ‘physical skills for occupational participation’ was one of the next frequently cited interventions and although typically used in a preliminary phase of the occupational therapy process, participants from a range of services appeared to identify ‘assessment’ as an intervention repeatedly. Other interventions categorised such as ‘facilitating access to resources’, the category of ‘prevention/maintaining health & well-being’, ‘modifying the environment’ and ‘cognitive skills for occupational participation’, ‘falls intervention’ and ‘occupations’ were also reported.

### Framework analysis findings

**Figure 4.**
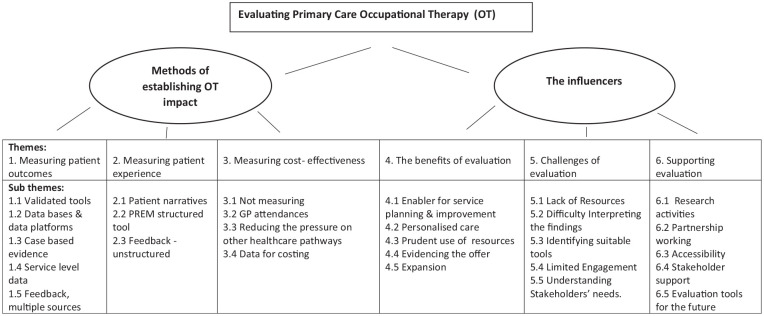
Thematic analysis framework.

Through synthesising data from across the qualitative Mentimeter responses and the eight questions that promoted focus group discussion, two main overarching themes were derived: methods of establishing occupational therapy impact and the influencers. These encapsulate evaluation methods being used and current perspectives and mechanisms that influence evaluation in practice through a number of further derived themes and subsequent sub-themes shown visually in the emergent conceptual framework (Figure 4). These are presented in turn and through using a selection of the anonymous Mentimeter response extracts (depicted in inverted commas) and condensed, direct unedited quotes taken from 9 out of the 13 participants present in the focus group who verbally participated during open discussion to substantiate the framework representation.

### Methods of establishing occupational therapy (OT) impact

The first overarching theme provides insight into the ways and means of evaluation that practitioners in occupational therapy services are currently using to determine their impact and potentially demonstrate value.

### Theme 1. Measuring outcomes

This category demonstrated how occupational therapists in Wales providing services to clusters are evaluating the impact of their service. Methods described facilitate this at an individual level as well as to demonstrate service level impact.

#### Validated tools

A small number of validated PROMs, Clinician-Rated Outcome Measures (CROMs) and a screening tool; the Patient Assessment Centered Assessment Method (Pratt et al., 2015) were reported in use. These varied from the clinician rated Therapy Outcome Measure (Enderby and John, 2015) to the Australian Occupational Therapy Outcome Measure-Occupational Therapy (AusTOM-OT; Unsworth and Duncombe, 2014) and to more globally administered quality of life measures such as the Recovering Quality of Life Measure (ReQol; Keetharuth et al., 2018) and EQ-5D-5L (The EuroQol group, 1990; Herdman et al., 2011). The use of AusTOMs was the most common tool cited by 9 out of the 12 participants who responded; however, there did not appear to be apparent patterns across types of setting or organisation and without any further consensus derived amongst the other PROMs tools in use.

#### Databases and platforms

This sub-theme included the ways and means through which occupational therapists are capturing and organising data to evidence their impact at all levels. Platforms to store and organise data, with participants identifying methods such as ‘team database’ and ‘GP database’ were identified. In addition, data types such as statistics as well as methods of collection for example through Microsoft Office or the use of a digital survey platform were discussed.

I’m a database convert. I used to hate databases, but I absolutely think that they’re brilliant now because every time I have to report the data, the data is live. And like you know I just had to do my last three months of report of the number of referrals and everything and it’s just so easy. It’s just there like that. And similarly with our patient feedback, we’ve now got access to CIVICA and I can actually get the reports about myself so it’s, it’s just makes life so easy. (Participant 6)

Throughout the discussion there appeared to be some sense of empowerment through the ownership of the data and having the skills to access it, potentially owing to the benefits of being able to use the data purposefully and with ease.

#### Case-based evidence

Half of the respondents reported the use of case studies in demonstrating the impact of their services. Whilst it was not entirely clear if these are being used locally or also shared more widely to demonstrate impact, this appeared a popular mechanism of evaluation for showcasing potential benefits.

We’ve used them loads to really prove our service. (Participant 7)

For all participants, the use of case-based examples were used alongside other methods of evaluation with indications that incorporating case-based evidence complimented the presentation and understanding of other types of evaluation data.

And we I think there’s it’s important to be able to give some narrative around data explain what it actually means a lot of the time. (Participant 3)

#### Service level data

Whilst closely linked to mechanisms identified previously, this sub-theme captured indicators to demonstrate collective impact of the service for use at a service level. Data collected outlined aspects of the service users’ journey throughout a care episode and responses included information about people accessing occupational therapy including; ‘referral data’. Suggestions for data relating to the outcomes of care were also provided such as: ‘patient contacts’; ‘intervention type’ and ‘alternative route (pathways)’.

#### Feedback, multiple sources

It appears occupational therapists are considering beyond what is captured directly from the receiver of care and creatively drawing on a range of resources including other team members, ‘staff feedback’ ‘MDT feedback’ and ‘GP feedback’ to establish the professions impact on supporting people’s health and well-being. Other feedback received included that from wider projects and went beyond the direct impact on people’s health to include levels of ‘staff satisfaction’ which were also included in this subcategory. This is indicative perhaps that during evaluation of the impact of the occupational therapy service, other benefits such as the professions impact on staff well-being are also being considered.

### Theme 2. Measuring patient experience

All participants identified at least one means of gathering feedback to evaluate aspects of patient experience with 7 of the 13 responses indicating the use of three or more methods. With the overall theme encapsulating methods for how patient experience specifically is considered in evaluation, a number of subcategories provided insight into the specific ways in which the participants are attempting to achieve this.

#### Patient narratives

The use of patient stories was the most popular method for denoting patient experience and indicated in just over half of responses. The use of case studies was also consistently popular to evidence this.

. . .Its getting the patient to think about their experience and how this differed as well from that just going to the GP having antibiotics as such and sent home. . . .And some of the feedback we’ve had has been absolutely amazing. We’ve turned them into case studies. We’ve used them as patient stories. We’ve used them loads to really prove our service. (Participant 7)

However, as one participant addresses, the method itself can on occasion be challenging when considering what is fit for purpose and representative when using patient feedback to communicate the emerging role of occupational therapy.

. . .the quality and maybe some of the information we’ve been able to use varies depending on the language that the patient may use. Like for instance, we had a fantastic story but he kept referring to us as occupational health and it’s like if your using that, we use it a lot for the service stuff that’s funded through the **** project. Yeah, it’s just not going to sit well. So it is really as much as powerful it can be. It has been really challenging to, to record the patient’s stories. . . (Participant 8).

#### PREM structured tool

Examples of structured, although unvalidated, measures were identified as being used in practice. Tools used included ‘bespoke questionnaires’, capturing service-specific feedback and often with support of a digital platform such as ‘CIVICA’ (CIVICA, 2023) which six out of 13 participants identified, albeit four of these participants were embedded in the same organisation.

#### Feedback – unstructured

It appeared from the data that in addition to more structured methods of collecting feedback, occupational therapists understand the importance of diversifying their means of capturing patient experience. Participants reported using a range of informal opportunities to collect feedback on their service, and this included evidence from ‘general discussions’; ‘word of mouth’ or ‘cards and letters’ which are being logged and used to evidence impact.

Anything we get fed back, whether it’s on the questionnaire, or just a nice little conversation, I had a text the other day off a relative saying this has really cheered my husband up so I was like take a photo of that and send that off. So there’s no formal channel. But we do keep a collection of it and save a copy of the questionnaire as well for future reference. (Participant 7)

### Theme 3. Measuring cost-effectiveness

In an attempt to establish current patterns of evaluation for primary care occupational therapists across Wales, this appeared the least well-considered area. Far fewer responses were made and attributed to the development of this theme with 3 out of the 13 respondents omitting to contribute to the Mentimeter question exploring this aspect of evaluation.

#### Not measuring

Some occupational therapists reported overtly that they do not consider costs when evaluating their service and some insight was provided into potential barriers. Participant 4 described that whilst in some aspects of the service, MDT level activity to support discharge can be costed using length of stay, for other areas of the service this is less well considered:

. . .When we got patients that we are marking as avoiding admission, we’re not measuring, we’re not seeing on average, they would have been in for x amount of time, because it didn’t reach that point, so there is this block to it on that, so I think from my perspective I’m not [measuring].

#### GP attendances

One way in which occupational therapists were attempting to establish cost-effectiveness, included the impact of the profession on GP attendances. This, in one reported case, enabled some simple cost analysis to be attempted:

The other thing I’m doing is looking at repeated tendencies at GP practices to try and look and see whether you know, if we do some input, do people come back less do they come back more what happens? And you can do some very rudimentary costings on that then. (Participant 3)

#### Reducing the pressure on other healthcare pathways

Apart from reducing pressure on GPs themselves, it was also suggested that cost benefits derived from occupational therapy input could be demonstrated from measuring benefits across the whole pathway of care provided. This included front-line emergency services, secondary care as well as for one practitioner who, by capturing reductions in home-based support packages, was able to demonstrate impact on social care:

I popped in about reductions in care packages. So in three of the clusters, what they’ve done, they’ve gone to brokerage and asked the individual cost of a care call cost per hour, made an average to get like that financial impact. (Participant 9).

#### Data for costing

Collecting data to potentially support cost analysis were identified and contribute to this theme. ‘Case study’ data as well as service level activity data such as ‘referrals’ and ‘stats’ were all regarded as a useful basis for measuring cost-effectiveness, albeit only a small number of the responses indicated this was being implemented in practice.

### The influencers

This overarching theme identified a range of themes and sub-themes that support an understanding of the influencing motivators, obstacles and opportunities that impact on evaluation in primary care practice. It also provides some insight into how drivers considered by occupational therapists align to principles of VBHC and in some cases support methods used.

### Theme 4. The benefits of evaluation

This theme provides some insight into the perceived advantages of evaluating occupational therapy in primary care. It includes benefits for the individual, the wider service and for the profession and suggests some understanding of the drivers for demonstrating both impact and value.

#### Enabler for service planning and improvement

A key motivator appeared to be the role of evaluation in service development. Evaluation appeared significant in a number of activities such as ‘service planning’ and ‘standards review’; however, what remained unclear was at what stage it was most important, for example did evaluation provide evidence leading to service improvements or were they perceiving evaluation as a useful mechanism to address the effectiveness of changes or developments made?

#### Personalised approach to care

This sub-theme encapsulates the importance of the patient or individual at the centre of their care and may suggest that practitioners recognise evaluation as an enabling mechanism for supporting person-centred practice. Aligning to both underpinning occupational therapy and VBHC implementation approaches, this was represented in a number of responses such as through ‘patient collaboration’ or ‘client centred’ and based on ‘understanding the need’, ‘identifying what works’ and ‘what matters most’. These responses denote an understanding of how evaluation and value-based approaches may support individual outcomes, and moreover with responses such as ‘better outcomes’; ‘added value’; ‘low cost’; ‘making a difference’ and ‘impact’, there may be recognition that the benefits of using person-focused evaluation methods could also contribute to demonstrating impact and achieving value at service, organisational and population health level.

#### Prudent use of resources

Despite the apparent lesser consideration for cost-effectiveness, this sub-theme suggests that practitioners recognise the benefit of demonstrating impact to inform more sustainable models of healthcare.

. . .I think it is one of those natural things that’s kind of embedded in, isn’t it? Like when we think about prudent healthcare, and where it’s originated from and things like Future Generations Act and all of these other aspects are things that have kind of made us or told us that we need to think differently about how we do things? Are we doing things efficiently? Are we doing things prudently? Are we doing things sustainably all of those sorts of aspects I think now, so embedded into the cultures of actually what we’re doing. . . (Participant 4)

Whilst it was not entirely clear whether the drivers to work more prudently is informing evaluation and demonstrating impact and value at a service or organisational level, with only one reference to the ‘health board’ in this process, the need to consider resource allocation was identified. Practitioners acknowledged the need for provision of the right service and within the right conditions to achieve ‘sustainable’ and ‘equitable’ healthcare, demonstrating understanding and alignment to the ambitions of a more value-based model of care.

#### Evidencing the offer

There appeared to be a strong consensus on the benefit of evaluating the service for broader purposes, particularly for promoting the value of what the profession can bring to primary care. In this sub-theme, there was a distinct focus on realising the importance of producing ‘what stakeholders need’. Whilst it was not clear specifically who this may include, there were several references to ‘demonstrating’ the contribution of the profession with a number of clear strategic purposes included, for example: ‘commissioning’; ‘benefits of embedding OT’ and developing the ‘evidence base’.

So we’re able to then use those sorts of reporting that we have at one level to be able to actually take up tiers and go this is what we could replicate in other areas around business cases. . . (Participant 4)

. . .I think our GP colleagues are using that feedback to understand our role better and understand the difference we’re making some of the really some of the complex people and what we’ve tried to do is reduce the barriers to its use as much as we can. . . (Participant 3)

The extracts included may suggest value is placed on evaluation for the needs of stakeholders who may oversee, plan and promote the role of occupational therapy in service delivery, rather than as in previous sub-themes, for those who use services.

#### Expansion

There appeared to be recognition for the possibility of ‘growth’ within the profession which may be potentially be a benefit of evaluation. This seemed dependent on resources such as ‘more staff’ required to develop provision such as ‘expansion in services’, ‘integration of teams’ and ‘cascading to rest of health board’. Whilst there was recognition of influences beyond the professions control, such as ‘cluster decisions’, there was also consideration for the enablers of this environment including ‘permanent funding’ and with some overlap to evidencing the offer, how collecting data may achieve this:

And he basically said that they were very impressed with the way that we collect data and show everything in our report, and that they’re thinking of funding my post, you know, being a Health Board funded post, so it’s not going to be one year yearly contracts. So which was quite nice to know that the data is gonna be used in the future and possibly used to secure the post. (Participant 6)

### Theme 5. Challenges of evaluation

Although the participants identified clear benefits which may motivate them to consider and implement evaluation, this theme provides insight into the difficulties in carrying out evaluation from their experience.

#### Lack of resources

The constraint of ‘time’ as a resource appeared to be a key limitation impacting on evaluation. Pressures such as ‘clinical responsibility’ and the ‘quantity required’ suggests that there is a perceived requirement for practitioners to spend time on gathering evaluation data themselves which is difficult to maintain. Other resources such as ‘support’ and ‘IT’ were also identified as potentially impacting on the ability to capture evaluation data as identified in other themes. This could suggest limitations may include the skill set of occupational therapists, difficulties with accessing support services or navigating IT provision in primary care, or simply not having the time to engage with them. Participant 7 was asked during discussion whether support was available to gather outcome data from people using services:

No, is the honest answer. We just upload it onto a database using a rating of one to five, one being the worst, five being the best, that then is created into a pie chart but then we also save on Excel we’ve got a free text box so we do all that narrative as well. The collection of the narratives so that then feeds into our um quarterly reports that go to Welsh government, so it tends to be the patient feedback bit which lets them [practitioners] down. (Participant 7)

Whilst gathering patient experience narratives are identified and potentially support evaluation, later sub-themes provide greater insight into difficulties associated with this.

#### Difficulty interpreting the findings

Challenges in using the data once gathered was identified. This appeared to be mostly attributed to the skills required both in data analysis to help ‘understand what it means’, and in ‘critically evaluating’ the data in order to present it.

#### Identifying suitable tools

Whilst evaluation tools for capturing data have been identified, it appears that there was consensus that measuring the impact of practice in this setting remains challenging. There appeared to be a perception that the right tool or ‘right outcome measure’ does not exist in this setting, and in one case this included specifically how to measure ‘cost’. Responses attributed this barrier to a ‘lack of suitable measures’; ‘best resource tools’ or ‘available tools’. When measures are available and used, further challenges are identified:

. . .So pre data with the outcome measure the pre outcome measure, fantastic, but it’s collecting a meaningful post outcome, because often we will only be able to collect that up maybe straight after the intervention and what we know is sometimes for our intervention it may take a little while to have that true impact. And, but what we also have to be mindful of is that there’s other things that can then impact that post score, even if we delayed it a little bit. So it’s trying to get something meaningful in a timely manner, I guess. (Participant 10)

Post-intervention, evaluation appears difficult to implement by some practitioners in the primary care setting due to timeliness and ‘sensitivity’ issues when using methods to capture the occupational therapy contribution.

#### Limited engagement

Participants also described challenges with engagement. These included both with staff and for those using the services. The role of staff in engaging with the process of evaluation was identified as problematic for some participants.

. . .Asking them for feedback still sits uncomfortably for a range of people when it’s very different to kind the black and white objective measure of doing an AUSTOMS or that functional measure, to be honest so I’ve definitely felt barriers in my team, getting it just because it just falls off the radar and isn’t something that people naturally automatically seem to think to do. (Participant 4)

Service user engagement with evaluation was also identified as the ‘response of population’ in this process; however, whilst later themes give some insight into the factors that impact on staff engagement, what was less clear from the data were potential contributors as to why those using services may be less engaged with evaluation.

#### Understanding stakeholders’ needs

A further barrier to evaluating occupational therapy practice in primary care was understanding what was needed to evidence the offer. There are potentially a number of stakeholders with an interest in the service provided and may include people who use services, as well as those who organise and provide them; however, the responses appeared to indicate this was considered mainly for the context of GPs and clusters themselves. This included a general lack of confidence in how best to present data in view of the ‘narrative needed’ and ‘language required’.

. . .we don’t report on everything but I pick out for my sort of yearly reports, you know, the bits that I wanted to, it’s not driven by, by the GPs or not really sure what they think about it. Never get much feedback, but then they there was they never say anything bad. So assume they’re quite happy. . . (Participant 6)

Despite uncertainty however, recognition of a general need to demonstrate the occupational therapy contribution was clearly identified.

### Theme 6. Supporting evaluation

Despite some of the challenges faced, this theme represents multiple mechanisms in place that enable primary care occupational therapists to consider evaluation.

#### Research activities

Activities across the research continuum were commonly identified as conducive to supporting evaluation. This included suggestions of: ‘audit’; ‘quality improvement’; ‘specific projects’; a national professional occupational therapy-specific evaluation and ‘research’ as enablers.

#### Partnership working

Throughout the discussions, the participants also raised examples of the value of working alongside and utilising the skills of other individuals and teams:

. . .its actually been a blessing being part of a bigger MDT model and have those costings done by project management team, they’ve got the time to really delve in look at the relevant figures and everything else and pull that off because for business cases and getting other staffing in it’s just been absolutely invaluable where other services are having to find cost savings, [our service] just keeps getting pump primed with money to do more. . . (Participant 4)

There was also evidence of how teamwork and communicating the purpose and results of evaluation can support this part of practice.

I think the difference now I know we spoke a bit about how it’s being captured, people using Microsoft Office wherever different tools on there, being able to show it up on an on a dashboard and demonstrating actually this is where the data is going, this is where it’s feeding into, goes a long way in terms of the barriers you may expect with staff. So I think within our team in particular, we’re all on board because we know that there’s a purpose behind it. (Participant 2)

Activities such as ‘staff engagement’, ‘team meetings’ and through raising an ‘awareness of purpose’, such as the example shown, provided insight into how engagement in evaluation can be facilitated. Another enabler was shared within the network group participants on how the process of evaluation has and continues to evolve.

So we will continuously look at are we collecting it? If we’re not, should we, if we are, should we? So every month I go through it with the team and just say look, we’re collecting this do we still need it? If we’re not reporting on right, are we ever going to report on it we’re not ever going to report on it and it’s not relevant, don’t collect it. . . (Participant 5)

There was also a strong sense that learning together through activities such as: ‘sharing methods’ and ‘benchmarking with others’ contributes positively to the development of evaluation. This included the potential of further developments such as ‘additional AHP staff’ within primary care Participant 2 identified:

it’s becoming a different kind of beast really and with the expectation that physio will be becoming part of the service, I’m interested to see what kind of joint outcomes from a therapies point of view are going to be considered and what kind of things will be used to capture that really.

Such development may impact on current practice or provide opportunities to support future occupational therapy evaluation.

#### Accessibility

Resources such as ‘technology’ and ‘databases’ were also identified as a key enablers pertinent to conducting evaluation. Whilst there may be some overlap with previous themes discussed, this sub-theme is more concerned with ensuring the technology and the platforms put in place are useable and accessible to aid engagement. As participants described, this includes both the occupational therapists and considerations for people accessing services they provide.

. . .I like to keep it simple. So we use Microsoft Excel. So to me that is simple and I’m, I’m always saying that the data needs to be relevant. . . (Participant 5)

So I’ve, we’ve made that as QR codes that people have printed out and gotten the surgery so people can scan it, and they can do the PREM. And I want it to be something that someone can do as they’re walking out the surgery. You know, I want it to be that quick and that easy. We’ve got it as a link on the base of our email. So whenever we’re sending stuff out to people its automatically there. I’m looking at getting iPads that we can have in surgery so that we can then just give to people and they can do the PREM on it. . . (Participant 3)

#### Stakeholder support

Finally, another key influence included stakeholders and reflects recognition for occupational therapy working alongside ‘higher level’ support mechanisms. Rather than a barrier as previously reported, stakeholders who consisted of people and organisations central to positively influencing this process included: ‘management’; ‘Welsh Government’ and ‘data sharing with GPs’. In contrast to previous observations of occupational therapists owning their data, a reference to ‘cluster ownership ‘of such resources was made.

I do it from, I have to do it for the cluster because I am sort of employed by them. But I know it’s been shared sort of higher up. . . (Participant 6)

This sub-theme may provide some initial insight into which stakeholders occupational therapists deem important to demonstrate impact to and how they achieve it.

That’s coming back to the conversation about speaking language, three of the cluster primary care teams are co-located with the local authority. So in terms of bang for the buck of the impact their OTS are making, that’s the language that they wanted. So that’s very much measured and that’s been incorporated into those clusters spreadsheet. (Participant 9)

#### Evaluation tools for the future

The final subcategory considers how tools for evaluation may need further exploration to support evaluation in the future. This subcategory has overlap with a previous theme exploring barriers. Suggestions included ‘national measures’, ‘consistent measures’ and for ‘more focused’ methods, indicate a lack of confidence in measures currently in use and with anticipation of further challenges.

I was gonna mention I really was very interested, I don’t know if anyone else saw it, there was a presentation where they were talking about a [new measure], which would have captured lots of the different measures that people have put up on the menti, all in one place. So I’m really interested in moving towards using some of that, but I think this is something that is it’s a really hard one to get into. (Participant 3)

Whilst it is recognised that identifying suitable tools for evaluation is not easy, the extract highlights the current motivation to find better ways of evaluating and evidencing the offer.

## Discussion

This is the first study to explore alignment of occupational therapy evaluation in primary care cluster settings to value-based healthcare. Representation from nearly all areas across Wales demonstrates the increasing visibility of occupational therapy in this setting. However, whilst occupational therapists have been reported to have the skills to work in primary care with individuals of all ages (Eichler and Keptner, 2023), in this study participants provided services exclusively to adults. This included addressing the needs of older adult populations in all cases, a finding consistent with other authors (Bolt et al., 2019; Donnelly et al., 2016, 2023) and is perhaps unsurprising given the concerns for an ageing population and the impact on healthcare. Similarly, the present study found services are commonly being offered to those with a combination of both mental health and physical needs. This is in keeping with a recent scoping review of the evidence for occupational therapy primary care services (Donnelly et al., 2023) and indicative that practitioners are keeping their offer broad to enable primary care providers to realise the potential of the profession (Bolt et al., 2019). Although occupational therapists have the potential to support accessibility to services for all age groups and populations at an early stage (Wood and Serfas, 2023), the findings suggest this is not presently on offer in clusters in Wales and reinforces that occupational therapists are predominantly undertaking chronic disease management most commonly with adults and supports other research (Donnelly et al., 2023). It could be argued that this remains a missed opportunity or certainly an area with potential for more consideration. However, given the ongoing agenda for chronic disease management using person-centred approaches (Bedlington et al., 2021), it remains logical that occupational therapists continue to draw on a range of interventions and adopt the more generalist approach, regarded as emblematic in primary care (Bolt et al., 2019; Donnelly et al., 2014, 2016, 2023; Muir, 2012). What remains unclear is how this broad agenda impacts on establishing value and impact through outcome evaluation and to address this further, the findings of the framework analysis are discussed within the context of VBHC.

### Establishing better population health

From the present study, occupational therapists appear to be recognising the importance of collecting and measuring outcomes to inform care at a micro individual level but also with the motivation and commitment to demonstrate impact more widely. This aligns to requirements for establishing value in health (Bedlington et al., 2021; Lewis, 2022) and may be unsurprising given the professions call to increase the evidence base (Donnelly et al., 2014; Hand et al., 2022; Jordan and Halle, 2023; Muir, 2012) and calls to strengthen the profession through an increasing focus on providing data (England, 2021; Royal College of Occupational Therapists, 2021a). It is therefore reassuring that occupational therapy practitioners appear committed to this and are utilising supportive mechanisms to approach both evaluation and gathering data.

What is apparent however is the inconsistency in outcomes being measured. To provide meaningful care that supports the health and well-being of individuals and populations, a variety of outcomes are typically implemented (Bedlington et al., 2021); however, in the present study, there appeared to be limited consensus in methods of evaluation being used. PROMs support the measurement of effectiveness at an individual level and create data which could be used to capture impact across systems (Hand et al., 2022; Lewis, 2022). With the exception of using global health measures in two cases, the uptake of validated tools and specifically patient rated measures to evaluate outcomes within this study were low and mainly limited to capturing aspect of patient experience. This finding supports the apparent uncertainty about what tools and data are best and for whom. It is consistent with recommendations made from a previous study in primary care-based occupational therapy that attempted to explore the feasibility of integrating a set of PROMs (Hand et al., 2022). Whilst integrating PROMs were deemed necessary and also valued by the therapists who participated, the study highlighted ongoing challenges in the selection of appropriate measures for practitioners to use (Hand et al., 2022).

The limited use of tools to support outcomes and demonstrate value on the basis of what matters most to people could arguably be a missed opportunity for occupational therapy; however, focus group participants did report the use of a CROM. AusTOMS-OT, a CROM that takes into account personalised goals from individuals allowing therapists to choose relevant scales across four domains of health: impairment; activity limitation; participation and distress/well-being (Unsworth and Duncombe, 2014), was frequently used. Occupational therapy is founded upon a notion that the person receiving care is central to practice and therefore uses profession-specific tools and measures that uphold professional values but also align to patient-centred approaches in healthcare (Mroz et al., 2015). Tools that encourage shared decision-making and setting goals have been established as central to enabling individuals to achieving value (Lewis, 2022; Wong et al., 2022). Therefore, if occupational therapists use profession-specific measures in collaboration with those using their services, they align to this agenda. These findings however are not entirely consistent with a recently conducted scoping review (Ingham et al., 2024). This review identified a range of measures including PROMs used routinely in occupational therapy primary care evaluation including one profession-specific tool, the Canadian Occupational Performance Measure (Law et al., 2014). The AusTOMS-OT measure however was not identified across the 16 international studies included. This suggests that whilst occupational therapists may be showing consideration for measures that align to the values of the profession and support outcomes synonymous with maintaining value in health, as yet there remains little consistency in the measures being selected.

Brief episodes of care provision and challenges in scheduling timely follow up to support effective post-intervention evaluation, including through the use of tools, are difficulties consistently reported in primary care occupational therapy (Donnelly et al., 2017; Hand et al., 2022). Given that ‘assessments’ were identified as an intervention by practitioners in the present study, the extent of occupational therapy input on occasions, whilst potentially still supporting health in this setting, may continuing to be present further challenges to demonstrating the contribution of the profession. These factors combined with the wide scope of occupational therapy roles identified both within the present study and wider practice (Bolt et al., 2019; Donnelly et al., 2023) may go some way to explain current inconsistency. Enablers identified, such as technology, may provide further opportunities for value-based evaluation and enhance data collection (Lewis, 2022) and systems such as those for capturing electronic medical records could warrant more exploration for their potential to support data management. However, further limitations such as time and a suitable skill set observed in the present study are also barriers consistent with wider AHP literature (Davenport and Underhill 2023; Duncan and Murray, 2012). These challenges may continue to impact on implementation of evaluation approaches, particularly when it comes to the requirement of providing data at scale. Despite this, what remains encouraging is that where available, practitioners are drawing on a range of resources to support them in the quest to learn about and develop ways in which to evaluate, capture outcomes and determine value. This is consistent with practitioner insight derived from previous studies (Hand et al., 2022; Wong et al., 2022) however upholds that more can be done. There was recognition that occupational therapists lack confidence in what stakeholders need and want from data and in particular validated measures. To support occupational therapists in their understanding of how best to approach outcome evaluation, in agreement with previous studies (Donnelly et al., 2017; Hand et al., 2022), further research to support PROM implementation and streamline evaluation data being collected for use in primary care is needed. Given the plethora of CROMS, PROMs and other sources of data available, further work could focus on identifying what stakeholders, who plan and commission services, need from evaluation of occupational therapy in primary care and how best they can support it. Furthermore, given the acknowledgement of the barrier of engagement in the study, there is a risk that people who use services may not find outcome measurement accessible (Duncan and Murray, 2012). To inform a better understanding of this, further research needs to ask all stakeholders how they want occupational therapists to capture outcomes to evidence impact.

### Improving experiences of care

The quality of care provided is one key aspect of value-based healthcare that occupational therapists appeared to be considering thoroughly. Using data to understand the experiences of those using services, through examining levels of satisfaction, is regarded as essential in ensuring healthcare quality (Custer et al., 2015; Sarsak, 2022). Whilst variation in measures to capture this were also observed in the current study, more consistency in the ways data were being collected directly from people using occupational therapy services was noted using both formal and less structured means. Previous studies that have considered how services implement measures to address quality and patient experience and albeit at population rather than at an individual level have reported conflicting results. Hendrikx et al., (2016) reported less consideration for patient perspectives within measurements implemented to evaluate value-based healthcare ambitions internationally, whereas more consideration for patient experience was reported in a review of value-based framework implementation in the context of primary care (Obucina et al., 2018). A recent Kings Fund report has suggested that to improve outcomes, greater consideration of patient experience is required (Wellings and Tiratelli, 2023). It appears in fact that occupational therapist are ahead of this agenda. Maybe given its relevance to providing a new model of care which has included the expansion of the profession into primary care, and through the professions person-centred approach which routinely includes involving people in all aspects of their care (Mroz et al., 2015), occupational therapists are aligned to considering patient experience readily and are using it for quality assurance in their practice.

### Better value

A key finding of the study was that cost of provision was the least well-considered aspect of evaluation. Similar to evaluating other aspects of value, the present occupational therapy skill set may have limited participant’s insight into tools or ways to measure this aspect of effectiveness at an individual or service level, compounded with a limited understanding of what is needed by stakeholders. This may also have consequences for gathering data at scale and understanding the professions contribution, as to achieve value a consideration of all denominators of value, including cost is required (Porter, 2010). Some evidence of how occupational therapists are attempting to consider this innovatively was gathered and methods, such as reducing pathways to other services, could be useful however to assess the professions contribution to achieving value, wider consideration of resource use and cost-effectiveness may be required. Challenges in determining this aspect of value in health are not exclusive to occupational therapy. Globally funding arrangements for healthcare vary. A review of the implementation of the value-based system revealed inconsistencies in evaluating cost-effectiveness worldwide and information technology and measuring cost across the whole patient pathway were highlighted as areas for further development (Mjåset et al., 2020). Furthermore, in the UK primary care context, a paucity of evidence has also highlighted the need for more research into cost-effectiveness (Thorn et al., 2020). For occupational therapy specifically, amidst current financial pressures, findings from a systematic review highlighted that to support the cost-effectiveness of occupational therapy far more attention to this area of evaluation is needed (Green and Lambert, 2016). Despite this, relatively few examples of cost-effectiveness analysis in primary care appear in the literature (Cook and Howe, 2003; Gillespie et al., 2022; Lambert et al., 2010). This taken together with the importance of cost to determine value, which in Wong et al.’s (2022) exploratory study, was not limited to monetary value but identified by people who use occupational therapy services to also include personal indirect costs, it appears that this remains an area of evaluation for development. Through drawing on resources cited by participants to support evaluation such as through benchmarking or partnership working, utilising other services more equipped with health economy or population health expertise, occupational therapists could go some way to achieve this. However, understanding what is needed from those that manage and organise care may also be key to a better understanding. Further research could focus on asking what stakeholders require from occupational therapy services to assist them in this aspect of evaluation to inform future primary care development and planning in population health.

### Limitations

This study was limited by its single focus group design. Whilst conducted in a larger group than is typically reported for focus groups (Jacobsen, 2021), given the pressures in practice and the network membership from across Wales, it was regarded as the least burdensome way of exploring a range of views. A further limitation could be the observation that not all members present in the focus group participated consistently. This may have contributed to over representation from some participants, however, to negate barriers for online contribution the Mentimeter poll supported participation.

It could also be suggested that the findings of this study are limited to occupational therapists practising in services in Wales. Whilst international variations of primary care models in service provision pose challenges to implementing research, some findings may have application to occupational therapists or AHPs working in other primary care contexts and are considering how to demonstrate the value and impact of their services. Further research could explore evaluation methods used by occupational therapists in other models of primary care provision.

Limitations could also include the chosen method of framework analysis and initially deductive approach supported by priori questions which could potentially limit the potential for a deeper understanding of the data. Framework analysis was chosen due to its flexible application to the multiple types of data collected in the focus group and to ensure the objectives of study were met through clearly identifying evaluation methods in practice through flexibly using both deductive and inductive analysis.

## Conclusion and implications

This study has explored evaluation methods for establishing occupational therapy impact through a consideration for patient outcomes, patient experience and cost-effectiveness. The influencers (benefits, challenges and supports) that impact on evaluating occupational therapy in primary care have also been identified and from this insight into methods being used to evaluate adult services by occupational therapists working within clusters in Wales has been derived. The range of methods observed is reflective of the combination of services being provided and supports an ongoing generalist approach. Whilst the values of the occupational therapy profession align to and appear to be supporting value-based healthcare ambitions in practice, there are current challenges which impact on evaluating outcomes and the professions ability to contribute to this approach.

Generally, a multifaceted approach to evaluation is reassuringly being used; however, inconsistency in methods used and less consideration for the denominator of cost-effectiveness potentially pose a risk to gathering data at scale. Further research is needed to support occupational therapists in determining suitable measures that can support a more consistent approach to capturing outcomes. To inform this, a greater understanding of what is needed from evaluation using insight from all stakeholders is required. A future focus of the authors research will include qualitative interviews with OT service planners as well as with those who use the services. Furthermore, children and younger people seem underrepresented in PCOT services currently; therefore, further research could examine current models of care for this group to determine how this groups needs are being catered for within current population health ambitions and if evaluation methods should in this area be a future focus of enquiry.

**Key findings**

- In Wales, occupational therapists understand the importance of evaluating practice and supported by professional values are aligning to quadruple aim ambitions when considering how to evidence the impact of their offer. Although this is being supported and done through a variety of methods, occupational therapists appear to be unclear about what stakeholders want and need to demonstrate the value of what their services are providing in primary care.

**What this study has added**

This study provides insight into the ways in which occupational therapists are considering evaluation in practice. It demonstrates that the values of occupational therapy align with the ambitions for a more sustainable value-based healthcare system, however also suggests that a better understanding of what stakeholders require from evaluation is needed.

## Supplemental Material

sj-docx-1-bjo-10.1177_03080226251320185 – Supplemental material for Primary care occupational therapist’s methods of outcome evaluation: Do they align to value-based healthcare? Supplemental material, sj-docx-1-bjo-10.1177_03080226251320185 for Primary care occupational therapist’s methods of outcome evaluation: Do they align to value-based healthcare? by Laura Ingham, Alison Cooper and Catherine Purcell in British Journal of Occupational Therapy

sj-docx-2-bjo-10.1177_03080226251320185 – Supplemental material for Primary care occupational therapist’s methods of outcome evaluation: Do they align to value-based healthcare? Supplemental material, sj-docx-2-bjo-10.1177_03080226251320185 for Primary care occupational therapist’s methods of outcome evaluation: Do they align to value-based healthcare? by Laura Ingham, Alison Cooper and Catherine Purcell in British Journal of Occupational Therapy

## References

[bibr1-03080226251320185] Atala M Bennington M Domholdt E (2025) Population-based practice in occupational therapy. Occupational Therapy in Health Care 39: 162–176. 37534451 10.1080/07380577.2023.2243515

[bibr2-03080226251320185] Bedlington N Kelley T Kidanemariam M , et al. (2021) Person-centred value-based health care. Available at: https://www.europeanallianceforvalueinhealth.eu/publication/person-centred-value-based-health-care/ (accessed 8 November 2023).

[bibr3-03080226251320185] Berwick D Nolan T Whittington J (2008) The triple aim: Care, health, and cost. Health Affairs 27: 759–769. 18474969 10.1377/hlthaff.27.3.759

[bibr4-03080226251320185] Bodenheimer T Sinsky C (2014) From triple to quadruple aim: Care of the patient requires care of the provider. The Annals of Family Medicine 12: 573. 25384822 10.1370/afm.1713 PMC4226781

[bibr5-03080226251320185] Bolt M Ikking T Baaijen R , et al. (2019) Occupational therapy and primary care. Primary Health Care Research & Development 20: e27. 10.1017/S1463423618000452 PMC6476805 32799974

[bibr6-03080226251320185] Braveman B (2015) Population health and occupational therapy. American Journal of Occupational Therapy 70: 1–6. 10.5014/ajot.2016.701002 26709420

[bibr7-03080226251320185] Brooks R Milligan J White A (2017) Sustainability and transformation plans: Occupational therapists and physiotherapists can support GPs. British Journal of General Practice 67: 525. 10.3399/bjgp17X693413 PMC5647912 29074696

[bibr8-03080226251320185] CIVICA (2023) CIVICA – All Uk Insights. Available at: https://www.civica.com/en-gb/insight/all-uk-insights/ (accessed 12 December 2023).

[bibr9-03080226251320185] Cook S Howe A (2003) Engaging people with enduring psychotic conditions in primary mental health care and occupational therapy. British Association of Occupational Therapists 66: 236–246.

[bibr10-03080226251320185] Custer M Huebner R Howell D (2015) Factors predicting client satisfaction in occupational therapy and rehabilitation. The American Journal of Occupational Therapy 69: 6901290040p6901290041–6901290069. 10.5014/ajot.2015.013094 25553753

[bibr11-03080226251320185] Dahl-Popolizio S Doyle S Wade S (2018) The role of primary health care in achieving global healthcare goals: Highlighting the potential contribution of occupational therapy. World Federation of Occupational Therapists Bulletin 74:8–16.

[bibr12-03080226251320185] Davenport S Underhill A (2023) The use of outcome measures and factors affecting use in adult social care occupational therapy services in the UK. Irish Journal of Occupational Therapy 51: 42–51.

[bibr13-03080226251320185] deNoyelles A Reyes-Foster B (2015) Using word clouds in online discussions to support critical thinking and engagement. Online learning (Newburyport, Mass.) 19(4). Available at: https://www.semanticscholar.org/paper/Using-Word-Clouds-in-Online-Discussions-to-Support-deNoyelles-Reyes-Foster/a3fc792040e60cbe3d095a98b6cafce94d6b9594 (accessed 11 August 2024).

[bibr14-03080226251320185] Department of Health (2016) Health and Wellbeing 2026: Delivering Together. Belfast: Northern Ireland Department of Health. Available at: https://www.health-ni.gov.uk/sites/default/files/publications/health/health-and-wellbeing-2026-delivering-together.pdf (accessed 14 January 2023).

[bibr15-03080226251320185] Domas White M Marsh E (2006) Content analysis: A flexible methodology. Library Trends 55: 22–45.

[bibr16-03080226251320185] Donnelly C Brenchley C Crawford C , et al. (2014) The emerging role of occupational therapy in primary care: Le nouveau rôle de l’ergothérapie dans les soins primaires. Canadian Journal of Occupational Therapy 81: 51–61. 10.1177/0008417414520683 24783488

[bibr17-03080226251320185] Donnelly C Leclair L Hand C , et al. (2023) Occupational therapy services in primary care: A scoping review. Primary Health Care Research & Development 24: e7–e7. 10.1017/S1463423622000123 PMC9884533 36617849

[bibr18-03080226251320185] Donnelly C Leclair L Wener P , et al. (2016) Occupational therapy in primary care: Results from a national survey. Canadian Journal of Occupational Therapy 83: 135–142. 10.1177/0008417416637186 27074910

[bibr19-03080226251320185] Donnelly C O’Neill C Bauer M , et al. (2017) Canadian Occupational Performance Measure (COPM) in primary care: A profile of practice. American Journal of Occupational Therapy 71: 1–8. 10.5014/ajot.2017.020008 29135432

[bibr20-03080226251320185] Duncan E Murray J (2012) The barriers and facilitators to routine outcome Measurement by allied health professionals in practice: A systematic review. BMC Health Services Research 12: 96. 22506982 10.1186/1472-6963-12-96 PMC3358245

[bibr21-03080226251320185] Eichler R Keptner K (2023) Transitions across the lifespan. In: Dahl-Popolizio S Smith K Day M , et al. (eds), Primary Care Occupational Therapy. Cham, Switzerland: Springer, pp. 69–78.

[bibr22-03080226251320185] Enderby P John A (2015) Therapy Outcome Measure for Rehabilitation Professionals, 3rd edn. Croydon, UK: J & R Press.

[bibr23-03080226251320185] England S (2021) Occupational therapy data – the most valuable commodity? British Journal of Occupational Therapy 84: 733–734.

[bibr24-03080226251320185] Gillespie P Hobbins A O’Toole L , et al. (2022) Cost-effectiveness of an occupational therapy-led self-management support programme for multimorbidity in primary care. Family Practice 39: 826–833. 35137039 10.1093/fampra/cmac006 PMC9508868

[bibr25-03080226251320185] Goldsmith L (2021) Using framework analysis in applied qualitative research. Qualitative Report 26: 2061–2076.

[bibr26-03080226251320185] Gray M (2015) The ‘triple value agenda’ must be our focus this century. NHS Confederation. Available at: https://www.nhsconfed.org/articles/triple-value-agenda-must-be-our-focus-century (accessed 10 November 2023).

[bibr27-03080226251320185] Green S Lambert R (2016) A systematic review of health economic evaluations in occupational therapy. British Journal of Occupational Therapy 80: 5–19.

[bibr28-03080226251320185] Hand C Donnelly C Bobbette N , et al. (2022) Examining utility and feasibility of implementing patient-reported outcome measures in occupational therapy primary care practice. British Journal of Occupational Therapy 85: 477–486. 40337131 10.1177/03080226211042272 PMC12033716

[bibr29-03080226251320185] Hendrikx R Drewes H Spreeuwenberg M , et al. (2016) Which Triple Aim related measures are being used to evaluate population management initiatives? An international comparative analysis. Health Policy 120: 471–485. 27066729 10.1016/j.healthpol.2016.03.008

[bibr30-03080226251320185] Herdman M Gudex C Lloyd A , et al. (2011) Development and preliminary testing of the new five-level version of EQ-5D (EQ-5D-5L). Quality of Life Research 20: 1727–1736. 21479777 10.1007/s11136-011-9903-x PMC3220807

[bibr31-03080226251320185] Hurst L Mahtani K Pluddemann A , et al. (2019) Defining value-based Healthcare in the NHS. CEMB Report Centre for Evidence-Based Medicine, University Of Oxford, UK, March.

[bibr32-03080226251320185] Ingham L Cooper A Edwards D , et al. (2024) Value-based outcome evaluation methods used by occupational therapists in primary care: A scoping review. JBI Evidence Synthesis 23(1): 108–142 10.11124/JBIES-23-00183 39506871

[bibr33-03080226251320185] Jacobsen K (2021) Introduction to Health Research Methods: A Practical Guide, 3rd edn. Burlington, MA: Jones & Bartlett Learning.

[bibr34-03080226251320185] Jordan K Halle A (2023) Administrative and operational considerations. In: Dahl-Popolizio S Smith K Day M , et al. (eds) Primary Care Occupational Therapy. Cham, Switzerland: Springer, pp. 15–29.

[bibr35-03080226251320185] Keetharuth A Brazier J Connell J , et al. (2018) Recovering Quality of Life (ReQoL): A new generic self-reported outcome measure for use with people experiencing mental health difficulties. British Journal of Psychiatry 212: 42–49. 10.1192/bjp.2017.10 PMC6457165 29433611

[bibr36-03080226251320185] Kelly L Cordeiro M (2020) Three principles of pragmatism for research on organizational processes. Methodological Innovations 13: 2059799120937242.

[bibr37-03080226251320185] Lamb A Metzler C (2014) Defining the value of occupational therapy: A health policy lens on research and practice. American Journal of Occupational Therapy 68: 9–14. 10.5014/ajot.2014.681001 24367949

[bibr38-03080226251320185] Lambert R Lorgelly P Harvey I , et al. (2010) Cost-effectiveness analysis of an occupational therapy-led lifestyle approach and routine general practitioner’s care for panic disorder. Social Psychiatry and Psychiatric Epidemiology 45: 741–750. 19688282 10.1007/s00127-009-0114-5

[bibr39-03080226251320185] Law M Baptiste S Carswell A , et al. (2014) Canadian Occupational Performance Measure (COPM), 5th edn. Ottawa: CAOT Publications ACE.

[bibr40-03080226251320185] Leland N Crum K Phipps S , et al. (2015) Advancing the value and quality of occupational therapy in health service delivery. The American Journal of Occupational Therapy 69: 6901090010p6901090011–6901090010p6901090017. 10.5014/ajot.2015.691001 PMC4281704 25553739

[bibr41-03080226251320185] Letts L Donnelly C Hand C , et al. (2024) Research priority 5: Setting the stage for research on the impact of occupational therapy in primary care. The British Journal of Occupational Therapy 87: 267–269. 40337533 10.1177/03080226231225359 PMC12033674

[bibr42-03080226251320185] Lewis S (2019) Value-based healthcare – meeting the evolving needs. Australian Health Review 43: 485. 32171343 10.1071/AHv43n5_ED

[bibr43-03080226251320185] Lewis S (2022) Value-based healthcare: Is it the way forward? Future Healthcare Journal 9: 211–215. 36561818 10.7861/fhj.2022-0099 PMC9761467

[bibr44-03080226251320185] Mjåset C Ikram U Nagra N , et al. (2020) Value-based health care in four different health care systems. NEJM Catylyst 1.

[bibr45-03080226251320185] Mroz T Pitonyak J Fogelberg D , et al. (2015) Client Centeredness and Health Reform: Key Issues for Occupational Therapy. American Journal of Occupational Therapy 69: 6905090010p6905090011–6905090018. 10.5014/ajot.2015.695001 PMC4564793 26356651

[bibr46-03080226251320185] Muir S (2012) Occupational therapy in primary health care: We should be there. The American Journal of Occupational Therapy 66: 506–510. 22917116 10.5014/ajot.2012.665001

[bibr47-03080226251320185] NHS England (2019) Interim NHS People Plan. Available at: https://www.longtermplan.nhs.uk/publication/interim-nhs-people-plan/ (accessed 11 August 2024).

[bibr48-03080226251320185] NHS Scottland (2018) National health and social care workforce plan Part 3 – Improving workforce planning for primary care in Scotland. Scottish Government. Available at: https://www.gov.scot/publications/national-health-social-care-workforce-plan-part-3-improving-workforce/ (accessed 11 August 2024).

[bibr49-03080226251320185] Obucina M Harris N Fitzgerald J , et al. (2018) The application of triple aim framework in the context of primary healthcare: A systematic literature review. Health Policy 122: 900–907. 29935730 10.1016/j.healthpol.2018.06.006

[bibr50-03080226251320185] Office for Health Improvement & Disparities (2023) A consensus on health aging. Available at: https://www.gov.uk/government/publications/healthy-ageing-consensus-statement/a-consensus-on-healthy-ageing (accessed 9 August 2024).

[bibr51-03080226251320185] Patton M (2015) Qualitative Research & Evaluation Methods: Integrating Theory and Practice, 4th edn. Thousand Oaks, CA: Sage Publications, Inc.

[bibr52-03080226251320185] Porter M (2010) What is value in health care? New England Journal of Medicine 363: 2477–2481. 21142528 10.1056/NEJMp1011024

[bibr53-03080226251320185] Porter M Pabo E Lee T (2013) Redesigning primary care: A strategic vision to improve value by organizing around patients’ needs. Health Affairs 32: 516–525. 23459730 10.1377/hlthaff.2012.0961

[bibr54-03080226251320185] Pratt R Hibberd C Cameron I , et al. (2015) The Patient Centered Assessment Method (PCAM): Integrating the social dimensions of health into primary care. Journal of Comorbidity. 5: 110–119. 29090159 10.15256/joc.2015.5.35 PMC5636039

[bibr55-03080226251320185] Primary Care One (2021) Cluster working. Available at: https://primarycareone.nhs.wales/cluster-working/ (accessed 5 January 2024).

[bibr56-03080226251320185] Ritchie J Spencer L (1994) Qualitative data analysis for applied policy research. In: Bryman A (ed.), Analyzing qualitative data. London: Routledge, pp. 305–329.

[bibr57-03080226251320185] Royal College of Occupational Therapists (2021a) Data Innovation Strategy 2021-2023. Available at: https://www.rcot.co.uk/practice-resources/informatics (accessed 5 January 2024).

[bibr58-03080226251320185] Royal College of Occupational Therapists (2021b) Top 10 priorities for occupational therapy research in the UK. London: Royal College of Occupational Therapists. Available at: https://www.rcot.co.uk/top-10 (accessed 3 April 2023).

[bibr59-03080226251320185] Royal College of Occupational Therapists (2023) Primary care evaluation. Available at: https://www.rcot.co.uk/primary-care-evaluation (accessed 25 January 2024).

[bibr60-03080226251320185] Sarsak H (2022) Patient Satisfaction with Occupational Therapy Services for Wheeled Mobility and Seating Devices. Occupational Therapy in Health Care 38: 1–14. 10.1080/07380577.2022.2121992 36107489

[bibr61-03080226251320185] Scottish Government (2020) Primary care services. Available at: https://www.gov.scot/policies/primary-care-services/ (accessed 14 January 2024).

[bibr62-03080226251320185] Spencer L Ritchie J O’Conner W , et al. (2014) Analysis in practice. In: Ritchie J Lewis J Nicholls C , et al. (eds), Qualitative research practice: A Guide for Social Science Students and Researchers, 2nd edn. Los Angeles: Sage, pp. 295–345.

[bibr63-03080226251320185] Starfield B (1992) Primary Care: Concept, Evaluation, and Policy. Oxford: Oxford University Press.

[bibr64-03080226251320185] Teisberg E Wallace S O’Hara S (2020) Defining and implementing value-based health care: A strategic framework. Academic Medicine 95: 682–685. 31833857 10.1097/ACM.0000000000003122 PMC7185050

[bibr65-03080226251320185] The EuroQol group (1990) EuroQol – a new facility for the measurement of health-related quality of life. Health Policy 16: 199–208. 10109801 10.1016/0168-8510(90)90421-9

[bibr66-03080226251320185] Thinley S (2021) Health and care of an ageing population: Alignment of health and social systems to address the need. Journal of Health Management 23: 109–118.

[bibr67-03080226251320185] Thorn J Man M Chaplin K , et al. (2020) Cost-effectiveness of a patient-centred approach to managing multimorbidity in primary care: A pragmatic cluster randomised controlled trial. BMJ Open 10: e030110. 10.1136/bmjopen-2019-030110 PMC7044971 31959601

[bibr68-03080226251320185] Unsworth C Duncombe D (2014) AusTOMs for Occupational Therapy, 3rd edn. Melbourne, VIC: La Trobe University.

[bibr69-03080226251320185] Usher R Connolly D (2018) Primary care in Singapore: An occupational therapy perspective. Proceedings of Singapore Healthcare 28: 141–142.

[bibr70-03080226251320185] Ward D Furber C Tierney S , et al. (2013) Using framework analysis in nursing research: A worked example. Journal of Advanced Nursing 69: 2423–2431. 23517523 10.1111/jan.12127

[bibr71-03080226251320185] Welling D Tiratelli L (2023) Making Patient Experience a Priority. The Kings Fund: Available at: https://www.kingsfund.org.uk/publications/making-patient-experience-priority (accessed 7 January 2024).

[bibr72-03080226251320185] Welsh Government (2018) A healthier Wales: Our plan for health and social care. Cardiff: Welsh Governement. Available at: https://www.gov.wales/healthier-wales-long-term-plan-health-and-social-care (accessed 25 January 2024).

[bibr73-03080226251320185] Welsh Government (2020) Allied Health Professions Framework for Wales: Looking Forward Together. Cardiff: Available at: https://www.gov.wales/allied-health-professions-ahp-framework (accessed 25 January 2024).

[bibr74-03080226251320185] Withers K Palmer R Lewis S , et al. (2021) First steps in PROMs and PREMs collection in Wales as part of the prudent and value-based healthcare agenda. Quality of Life Research 30: 3157–3170. 33249539 10.1007/s11136-020-02711-2 PMC7700742

[bibr75-03080226251320185] Wong S Ngooi B Kwa F , et al. (2022) Exploring the meaning of value-based occupational therapy services from the perspectives of managers, therapists and clients. British Journal of Occupational Therapy 85: 377–386. 40337661 10.1177/03080226211030095 PMC12033699

[bibr76-03080226251320185] Wood J Serfas K (2023) Pediatric primary care. In: Dahl-Popolizio S Smith K Day M , et al. (eds), Primary Care Occupational Therapy. Cham, Switzerland: Springer, pp. 331–374.

[bibr77-03080226251320185] World Health Organization (2018) Building the primary health care workforce of the 21st century. Available at: https://apps.who.int/iris/handle/10665/328072 (accessed 9 August 2024).

[bibr78-03080226251320185] World Health Organization and United Nations Children’s Fund (2018) A vision for primary health care in the 21st century: Towards universal health coverage and the Sustainable Development Goals. Available at: https://iris.who.int/handle/10665/328065 (accessed 9 August 2024).

[bibr79-03080226251320185] World Health Organization and United Nations Children’s Fund (2020) Operational framework for primary health care: Transforming vision into action. Available at: https://www.who.int/publications/i/item/9789240017832 (accessed 9 August 2024).

