# Supplementary material for: Primary care occupational therapist’s methods of outcome evaluation: Do they align to value-based healthcare?
Source: Br J Occup Ther. 2025 Feb 24;88(6):362–78. doi: 10.1177/03080226251320185 (PMC12103676; doi:10.1177/03080226251320185)
Supplement: sj-docx-1-bjo-10.1177_03080226251320185 – Supplemental material for Primary care occupational therapist’s methods of outcome evaluation: Do they align to value-based healthcare? [file sj-docx-1-bjo-10.1177_03080226251320185.docx]

**Supplementary information (1):**

**Mentimeter and Focus Group Questions**

Thank you for agreeing to take part. Your participation is important to me and your opinions are extremely valuable.

Please could you confirm that you have been sent a participant information sheet which explains the aims of the research, and that you have read and understood it? *[Pause for confirmation].*

You have also been sent a link to provide e-consent for your participation, thank you. Please could anyone present who has not completed the e-consent form let me know so I can upload a link to the form in the chat box. *[Pause for confirmation]*

To remind you, as outlined in the participant information sheet, you are aware that this online focus group is being audio recorded using a password protected iPad and Otter software? This is to enable the content of our conversation to be transcribed for analysis. *[Pause for confirmation]*

All details of the discussion will be kept confidential; mentimeter is entirely anonymous and your name will be changed in the transcript to a pseudonym and when findings from this study are written up and published or presented at conferences it will not be possible to identify you. Throughout the discussion, please avoid disclosing any names or information that could identify an individual or organisation. If you do include anything that identifies someone or an organisation, this will be removed from the final transcript to maintain confidentiality and anonymity. Due to the nature of the interactive focus group, once we have begun it will not be possible to withdraw your data from the study.

Does anyone have any questions and are you happy to continue? *[Pause for questions/confirmation]*

*Pause….*

*Powerpoint….*

Please could you copy and paste the code in the chat into your search bar or if have a smart phone open up the QR code in the chat using your mobile phone/iPad.

Mentimeter question 1 (anonymous response)

What organisation do you work in (or if no longer working in primary care, which one did you work in)? List options for health boards, GP cluster employed or other.

Mentimeter question 2 (anonymous response)

Can you tell me the banding of your present role. If you are no longer in a primary care setting, please specify the band you were in when you were working there. (Options for student or band) **1 min**

Mentimeter question 3 (anonymous response)

How long have you been in (or were in) this role? List of options

Mentimeter question 4 (anonymous response)

Can you tell me about the type of service you provide in your setting? [*For any students present – ask about practice placement setting*]. List range of options

Mentimeter question 5 (anonymous response)

Can you tell me about your role in this setting by giving me an indication of the interventions you use? Word cloud

Mentimeter question 6 (anonymous response)

What age range do you work with? List of options

Mentimeter question 7 (anonymous response)

What do you understand about value-based healthcare? Word cloud

Any thing in particular that has supported this understanding, courses etc

Mentimeter question 8 (anonymous response)

**What evaluation methods do you use to establish if you have had impact on the health and wellbeing of the individuals you have worked with (Better Health)**

Word cloud then focus group open discussion

Prompt: **which tools?**

how is evaluation data collected, **who and what determines what is collected/evaluated**? How are these presented, who for?

Mentimeter question 9 (anonymous response)

**What evaluation methods do you use to explore patient experience?** **(patient experience)**

Word cloud then focus group open discussion

Prompt: how are these collected? who/how chooses what is collected? **how and who is this presented to?**

Mentimeter question 10 (anonymous response)

**What evaluation methods do you use to establish if your services have been cost effective**?

Word cloud then focus group open discussion

Prompt: how are these collected? who/how chooses what is collected? how and who is this presented to? **Any barriers or enablers to evaluating cost effectiveness**

Mentimeter question 11 (anonymous response)

**Are there any other evaluation methods routinely used or data collected to evidence the impact of your services?** (opportunity to add others)

Word cloud then focus group open discussion

Prompts: who chooses what is used? Is there any local support? how is data collated? who is it presented to?

Mentimeter question 12 (anonymous response)

What are the benefits of evaluating your services?

Word cloud then focus group open discussion

Prompts: what mechanisms support data collection? what works well? how is the data used?

Mentimeter question 13 (anonymous response)

What are the challenges of evaluating your services?

Word cloud then focus group open discussion

Prompts: what are the barriers to evaluating and collecting data? Time, skills, IT resources, support?

Mentimeter question 14 (anonymous response)

**What supports evaluation of OT services in primary care in your service**?

Word cloud then focus group open discussion

Prompts: what would help support evaluation? **What would you like to see in place to support evaluation of your service**; are there any things that could assist you?

Mentimeter question 15 (anonymous response)

Thinking about the future, do you envisage any changes to the services you provide and how would you like to see them evaluated?

Word cloud then focus group open discussion

Prompts: What changes do you anticipate? how would you like to evaluate them? what would effective evaluation look like?

Thank you for your time today, your participation is greatly appreciated. I will be checking the Otter transcription against the audio recording made and I will contact you so that you can review the transcript and inform me of any changes required. I may also contact you if I need you to clarify anything that you have said today.

After this focus group I’ll be emailing a Debrief Sheet to you straight away, explaining what will happen to your information now and where you can go to access support should you need to.

Thank you once again.

****END RECORDING*

[Email Debrief Sheet immediately after focus group]
