# Supplementary material for: Primary care occupational therapist’s methods of outcome evaluation: Do they align to value-based healthcare?
Source: Br J Occup Ther. 2025 Feb 24;88(6):362–78. doi: 10.1177/03080226251320185 (PMC12103676; doi:10.1177/03080226251320185)
Supplement: sj-docx-2-bjo-10.1177_03080226251320185 – Supplemental material for Primary care occupational therapist’s methods of outcome evaluation: Do they align to value-based healthcare? [file sj-docx-2-bjo-10.1177_03080226251320185.docx]

**Supplementary information (2): Example of content analysis - Interventions**

| Codes (Frequency of response): | Categories (frequency of codes included): |
| --- | --- |
| Self - management (7)  Equipment (4)  Relaxation (2)  Splinting (1)  Anxiety management (1)  Fatigue management (1)  Confidence building (1)  Joint Protection (1)  Sleep Hygeine (1)  Holistic Assessment (1)  Transfer (1)  Mobility (2)  Bathing (1)  External Assessments (1)  Advice and Signposting (6)  Goal Setting (1)  Education (2)  Shared Decision making (1)  3^rd^ Sector (1)  Falls assessment (2)  Falls prevention (1)  Falls work (4)  Adaptations (2)  Aids & adaptations (1)  Cognitive assessment (2)  Social prescribing (1)  Functional assessments (3)  Physical assessment (1)  Hand Exercises (1)  Short term rehabilitation (2)  Preventative (1)  Preventative & crisis work (1)  Chronic condition management (1)  Self help (1)  Well-being (1)  Carers Support (2)  Symptom management (1)  Technique (1)  ADLs (1) | **Self-management, symptom management (10)**  Self – management, Relaxation, Anxiety management,  Fatigue management, Joint Protection  Sleep Hygeine, Chronic condition management, Self help, Symptom management  **Modifying the environment (3)**  Equipment, Adaptations, Aids & adaptations  **Physical skills for occupational participation (5)**  Splinting, Transfer, Mobility, Hand Exercises, Technique  **Cognitive skills for occupational participation (1)**  Confidence building  **Assessment (5)**  Holistic Assessment, External Assessments, Cognitive assessments, Physical assessments, Functional assessments,  **Occupations (2)**  ADL’s, Bathing  **Facilitating access to resources (4)**  Advice and Signposting, Education, Social prescribing, 3^rd^ Sector    **Brief interventions (1)**  Short-Term rehabilitation  **Prevention/maintaining health & well-being (4)**  Preventative, Preventative & crisis work, Well-being, carers support  **Falls intervention (3)**  Falls assessment, falls prevention and falls work |
